# Identification of an Endogenous Strong Promoter in *Burkholderia* sp. JP2-270

**DOI:** 10.3390/microorganisms12091818

**Published:** 2024-09-02

**Authors:** Jing Ke, Jiamin Shen, Haoran Wang, Xinxin Zhang, Yucong Wang, Guoqing Chen, Guozhong Feng

**Affiliations:** State Key Laboratory of Rice Biology and Breeding, China National Rice Research Institute, Hangzhou 310006, China; kejing139723@163.com (J.K.); 545809610@163.com (J.S.); wanghaoran_12@163.com (H.W.); zhangxinxin_19@163.com (X.Z.); yucongwang2000@yeah.net (Y.W.); chenguoqing@caas.cn (G.C.)

**Keywords:** *Burkholderia* sp. JP2-270, pyrrolnitrin, promoter, overexpression, RNA-Seq

## Abstract

*Burkholderia* is the second largest source of natural product bacteria after *Actinomyces* and can produce many secondary metabolites including pyrrolnitrin (PRN). Natural products of microbial origin are usually found in trace amounts, so in metabolic engineering, promoter engineering is often used to regulate gene expression to increase yield. In this study, an endogenous strong promoter was identified based on RNA-seq to overexpress biosynthetic genes to increase the production of PRN. By analyzing the transcriptomic data of the antagonistic bacterium *Burkholderia* sp. JP2-270 in three different development periods, we screened 50 endogenous promoters with high transcriptional activity, nine of which were verified by an obvious fluorescent signal via fluorescence observation. Then, combined with RT-qPCR analysis, P*_hp_*, the promoter of a hypothetical protein, was found to be significantly expressed in all three periods. In order to increase the suitability of endogenous promoters, the promoter P*_hp_* was shortened at different lengths, and the results show that a sequence length of 173 bp was necessary for its activity. Moreover, this promoter was used to overexpress the PRN biosynthesis genes (*prnA*, *prnB*, *prnC* and *prnD*) in JP2-270, resulting in a successful increase in gene expression levels by 40–80 times. Only the overexpression of the *prnB* gene successfully increased PRN production to 1.46 times that of the wild type. Overall, the endogenous strong promoters screened in this study can improve gene expression and increase the production of secondary metabolites in JP2-270 and other strains.

## 1. Introduction

*Rhizoctonia solani* is an important fungal pathogen and causes rice sheath blight, which can lead to reductions in rice yield [1,2]. The application of fungicides is the most widely used method for controlling rice sheath blight. Validamycin is an agricultural antibiotic that once made a significant contribution to the prevention and control of the disease [3]. However, in recent years, there has been a growing number of local reports of rice sheath blight’s resistance to validamycin [4]. Therefore, it is necessary to develop alternative biocontrol agents. Many studies have reported the natural activity of some fungi and bacteria against fungal pathogens, which is considered a very attractive alternative to chemical fungicides [5,6].

*Burkholderia* exists widely in soil and other ecological environments, and it is the second major class of bacteria as a source of natural products after *Actinomycetes* [7]. Many studies have reported that a wide range of secondary metabolites is produced by *Burkholderia*, including pyrrolnitrin, phenazine, cepabactin, and other volatile compounds [8,9,10]. Most of these compounds have inhibitory activities against plant pathogens, including *Ralstonia solanacearum*, *R. solani*, and *Verticillium dahlia*, among others [11]. Pyrrolnitrin [PRN, 3-chloro-4-(2-nitro-3-chlorophenyl) pyrrole] was originally purified from *B. pyrrocinia* 2327T, and it has been proven to have good antifungal activity [12]. Although PRN can be obtained via chemical synthesis, this method increases the cost of the synthesis route and poses a threat to the environment [13]. Therefore, there is a preference for selecting microbial species as they offer a more selective, environmentally friendly and cost-effective method of synthesizing PRN.

Metabolic engineering is often used to regulate and optimize metabolic flux [14,15]. Promoter engineering is considered one of the most effective strategies for fine-tuning transcriptional control and has been successfully applied to many model microorganisms [16]. The promoters P_2057_ and P_2703_ were screened from *Gluconobacter oxydans* for the overexpression of the SDH dehydrogenase gene, which increased the production of 2-KLG [17]. Promoter strength screening is based on high mRNA expression in the transcriptome [18]. Based on RNA-Seq analysis, ten strong promoters and four component promoters were identified from *Streptomyces albus* J1074. These promoters can successfully activate a heterogenic hidden gene cluster in J1074, and they can also be used in three other widely used *Streptomyces* strains [19]. In recent years, studies have reported on the application of promoter engineering in *Burkholderia*. The strong component promoter screened from *B.* DSM 7029 can successfully drive the expression of foreign gene clusters in the strain [20]. When the commonly used rhamnose-induced promoter was modified for expressing foreign protein, the expression level of foreign protein was also improved in *B. cenocepacia* K56-2 [21].

*Burkholderia* sp. JP2-270 is a strain isolated from rice rhizosphere soil in our laboratory, which exhibits a good inhibitory effect on rice sheath blight, with the whole genome being sequenced. In this study, based on RNA-Seq analysis of JP2-270, a panel of promoters with different strengths was identified. These promoters were experimentally evaluated via the enhanced green fluorescent protein (eGFP) reporter and RT-qPCR in JP2-270. Furthermore, when the strongest screened endogenous promoter was used to overexpress the PRN biosynthesis genes, the production of PRN was 1.46 times higher than that of the wild type. Taken together, we screened an endogenous strong promoter that can significantly increase the yield of PRN by overexpressing biosynthetic gene in JP2-270.

## 2. Materials and Methods

### 2.1. Genes, Plasmids and Strains

*Escherichia coli* TOP10 was used for plasmid construction. The pathogenic fungus is *Rhizoctonia. solani* GD118. *Burkholderia* sp. JP2-270 (Genbank accession CP029824.1 to CP029828.1) was used for PCR amplification of gene and promoter sequences. *Burkholderia* sp. JP2-P*_hp173_*-*prnB* was used to produce PRN. The plasmid pBBR1MCS-Cm was derived from pBBR1MCS-2, and it was used to overexpress the *eGFP* gene and PRN biosynthesis-related genes in JP2-270. All strains and plasmids are listed in Table 1.

### 2.2. In Vitro Inhibition Assay

The Petri dish method was used to detect the in vitro antagonism of the fermented crude extracts of JP2-270 and its derivatives to *R. solani* GD118. The mycelium pieces (with negligible diameters) of *R. solani* GD118 were cultured for 48 h and then placed in the center of the plate. A volume of 15 μL of the fermented crude extract of overnight cultured JP2-270 and its derivatives was added to both sides, 2.5 cm away from the center of the PDA plate. After 2 days of co-culture, the bacteriostatic zone formed in the culture dish was determined to evaluate the bacteriostatic effect. The inhibition rate was calculated by using the average value of three biological replicates. Inhibition rate (%) was calculated using the following formula: % inhibition = [(C − T) × 100]/C, where C represents the fungal diameter (cm) in the control plate, and T represents the fungal diameter (cm) in the treatment plate [23].

### 2.3. RNA Sequencing (RNA-Seq) and Data Analysis

JP2-270 was cultured in LB medium at 30 °C with shaking at 220 rpm. According to the growth curve (Figure S1), the cells were collected at the exponential phase (16 h), stationary phase (24 h) and decline phase (48 h). Total RNA was extracted with RNAiso Plus (Takara, Japan). The qualified samples were sent to Beijing Novogene Corporation (Novogene, China) for transcriptome sequencing. The acquisition of transcript abundance is mainly accomplished by the following steps: FastQC (version 1.8.0_372) mainly examines the quality of raw sequencing data to obtain clean reads [24]; HISAT2 maps clean reads to the reference genome of JP2-270 [25]; SAMtools (version 1.3.1) sorts the results of the mapping from the previous step [26]; and StringTie quantifies reads on the reference genome and calculates the transcript per million value (TPM) [27]. Genes with high TPM values were also thought to have high transcription levels [28]. The highly expressed genes were selected according to the ranking of TPM values from high to low. The promoter prediction platform BDGP [https://www.fruitfly.org/seq_tools/promoter.html (accessed on 10 April 2023)] was used to search for regions with promoter sequence characteristics in the upstream region of genes. The RNA sequencing data were submitted to the Sequence Read Archive (SRA) with the accession number PRJNA1037687.

### 2.4. Construction of Gene Expression Vectors

To characterize the strength of endogenous promoter, a reporter expression vector was constructed with the pBBR1MCS-Cm expression vector and a reporter gene, *eGFP*. All screened potential promoters were amplified from the genomic DNA of JP2-270. All promoters work with the same natural ribosome binding site (RBS) (TAAGGAGGTTTTCTA) [29]. The *eGFP* gene was stored in our laboratory and obtained via PCR amplification. First, the fusion fragment of the promoter sequence and the *eGFP* gene was amplified via fusion PCR. Then, the fusion fragments of different promoters were inserted into the expression vector pBBR1MCS-Cm via a one-step cloning kit (Vazyme, China). Finally, the recombinant vectors were transferred via electroporation into JP2-270, which were selected using chloramphenicol. All primers are listed in Supplementary Materials, Table S1.

### 2.5. Real-Time Quantitative PCR

The promoter detection strains were cultivated in LB at 30 °C; then, the cells were collected at the exponential phase (16 h), stationary phase (24 h) and decline phase (48 h). Total RNA was extracted from overexpressed recombinant strains after being cultured for 24 h. mRNA was used as the template to reverse transcribe cDNA using the reverse transcription reagent HiScript III All-in-one RT SuperMix Perfect for qPCR (Vazyme). Real-time qPCR using a cDNA template was performed on a CFX96 Touch Deep Well real-time qPCR detection system (Bio-Rad, California, USA) using 2 × TB Green Premix Ex Taq II (TaKaRa). Using the *recA* gene as an internal reference gene, the relative transcription level of the gene was calculated using the 2^−ΔΔCt^ method [30].

### 2.6. Detection of Fluorescence Intensity

The promoter-detected strains were cultured in LB to a stable stage. Cells with the same OD_600_ value were collected, washed three times with sterile water, and then suspended in 100 μL of sterile water. A volume of 10 μL was added to the slide, and excess fluid was sucked away to stabilize the cells. A confocal laser scanning microscope LSM700 (Zeiss, Germany) was used to observe green fluorescence images utilizing an argon laser with an excitation wavelength of 488 nm. The actual fluorescence intensity was calculated by subtracting the background fluorescence signal of JP2-270 harboring pBBR1MCS-Cm.

### 2.7. Promoter Shortening

The BPROM website [http://www.softberry.com/berry.phtml?topic=bprom&group=help&subgroup=gfindb (accessed on 5 October 2023)] was used to predict the core components of P*_hp_*. The promoter sequence shortening method was carried out according to the site-directed, ligase-independent mutagenesis (SLIM) method [31]. The pBBR1MCS-Cm-P1-*eGFP* plasmid was used as the template. Each shortening reaction contained four primers, and pbbr1-f/pbbr1-r was used in all reactions; two other specific independent primers are listed below for each shortening reaction. The 1000 bp length of P*_hp_* was shortened to 500 bp with L500-f/L500-r primers, 349 bp with L349-f/L349-r primers, 173 bp with L137-f/L137-r primers, and 85 bp with L85-f/L85-r primers.

### 2.8. Shake-flask Fermentation for PRN Production via JP2-270

After the overexpressed recombinant strains were activated by plate scribing, single colonies were cultured in 5 mL liquid medium at 30 °C with shaking at 220 rpm overnight. The 1 mL overnight culture was inoculated in 100 mL of fermentation medium in a 500 mL flask. Samples with an initial cell density of OD_600_ = 2 were cultured at 30 °C, with shaking at 220 rpm for 120 h. After fermentation, the bacterial cells were collected via centrifugation at 12,000 rpm for 15 min. Ammonium sulfate was added to the resulting supernatant, stirred until completely dissolved, and kept at 4 °C overnight. After the precipitate was extracted with methanol, the organic phase was collected via centrifugation at 12,000 rpm for 15 min. Finally, the crude extract was dissolved in an 80% methanol solution after rotational evaporation.

### 2.9. High-Performance Liquid Chromatography (HPLC) Analysis

The crude extracts of the fermentation broth of overexpressed recombinant strains were filtered through a 0.22 μm filter membrane and then transferred into sample bottles. Agilent Technologies 1260 infinity (USA) was used for HPLC analysis. The chromatographic analysis was performed on an Agilent Eclips Plus C18 column with a particle size of 3.5 μm and 4.6 mm × 100 mm; chromatographic-grade acetonitrile–aqueous solution was used for the mobile phase; the flow rate of the mobile phase was 1.0 mL/min, and elution was performed for 30 min; the detection wavelength was 210 nm, and the sample size was 5 μL. The sequence program was run after setting the method and sequence according to the instrument’s instructions. The retention times of PRN in the given samples were then compared with the retention time of the standard PRN.

### 2.10. Statistical Analysis

The results were interpreted with mean values and the standard deviation of three independent experiments (*n* = 3). Statistical analysis was performed using Student’s *t-*test with GraphPad Prism software 8.0 to demonstrate statistically significant differences between data points. The *t*-test was used to compare different data and the results were considered to be statistically different when *p* < 0.05 and significantly different when *p* < 0.01. Significance is indicated in figures as follows: *, *p* < 0.05; **, *p* < 0.01; ***, *p* < 0.001; ****, *p* < 0.0001; and ns, not significant.

## 3. Results

### 3.1. Screening of Endogenous Strong Promoters of JP2-270 Based on Transcriptome Analysis and Promoter Prediction

To identify the strongly active promoters in JP2-270, the mRNAs isolated from the exponential phase (16 h), stationary phase (24 h) and decline phase (48 h), according to the growth curve of JP2-270 (Figure S1), were sequenced with high-throughput Illumina paired ends to obtain transcriptome data after removing ribosomal RNAs. Among the 8282 genes in the JP2-270 genome, about 1.4% of the genes had TPM values above 1000, about 18.6% of the genes had TPM values between 100 and 1000 and nearly 80% of the genes had TPM values below 100 (Figure S2).

To identify the strong promoter in JP2-270, we analyzed genes that exhibit strong transcriptional activity. Genes with the top 50 TPM values in the transcriptome data for each period were considered highly active at the transcriptional level (Tables S2–S4), and a total of 150 highly expressed genes were obtained. After removing the intergroup duplicate genes and genes coding for RNA, the remaining genes were selected to predict the promoter core region by selecting the sequence approximately 1000 bp upstream of the open reading frame (ORF). Finally, 50 sequences with promoter characteristics were obtained (Table S5).

### 3.2. Evaluation of Promoter Strength by Measuring eGFP Expression and RT-qPCR

**Figure 1:**
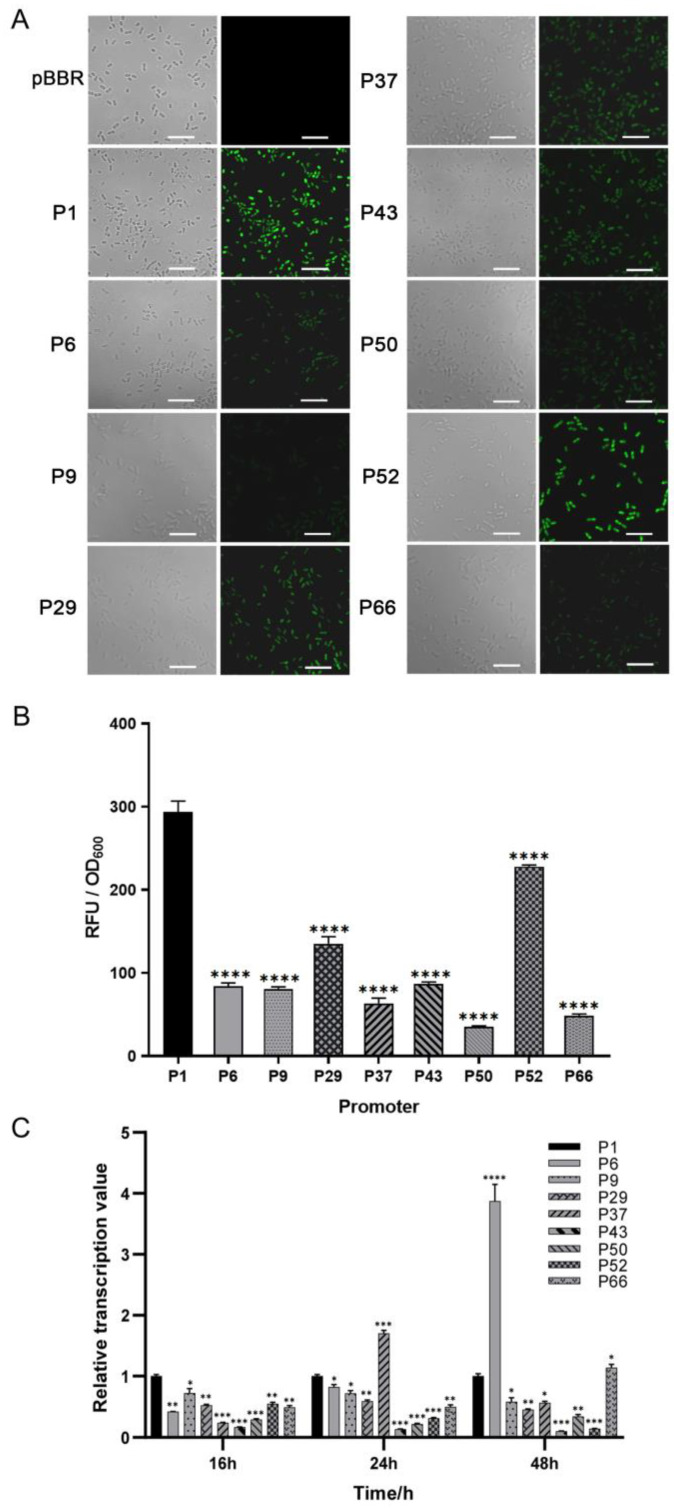
The strength determination of the screened promoters of *Burkholderia* sp. JP2-270. (**A**) The fluorescence intensity was detected via confocal microscopy. PBBR is the transfer of empty plasmids into JP2-270. P1, P6, P9, P29, P37, P43, P50, P52 and P66 are the 9 promoters that can detect green fluorescence. All images were obtained under the same detection conditions. Scale bar = 5 μm. (**B**) The fluorescence intensity was detected via confocal microscopy. (**C**) The 9 endogenous promoters that showed fluorescence were identified via RT-qPCR analysis. The transcription levels of the *eGFP* gene controlled by different promoters at different growth stages of JP2-270 were determined. The data represent the mean ± standard deviation of three repeated measurements from three independent experiments. Statistical analyses were performed and marked (*, *p* < 0.05; **, *p* < 0.01; ***, *p* < 0.001; and ****, *p* < 0.0001).

To mine endogenous strong promoters in the JP2-270 genome, we first examined the promoter strength at the translation level using laser confocal microscopy. Of the 50 candidate promoters screened based on RNA-Seq analysis, 9 promoters (P1, P6, P9, P29, P37, P43, P50, P52 and P66) were detected with significant fluorescence. Among them, the fluorescence intensity of P1 was significantly higher than that of the other promoters (Figure 1).

In order to further detect the activation effect of these nine promoters at the transcriptional level, we analyzed them via RT-qPCR at three different periods—16 h, 24 h and 48 h. The transcription level of P1 was set as the control. In the exponential phase, the transcription level of the P1 promoter was the highest. In the stationary phase, the transcription level of most other promoters, except for P6 and P37, was also lower than that of P1 (Figure 1C). P1 is the promoter of a hypothetical protein (DM992_24350), and, thus, it was renamed P*_hp_* and selected for further analysis.

### 3.3. Shortened Analysis of P1 (P_hp_) Promoter

**Figure 2:**
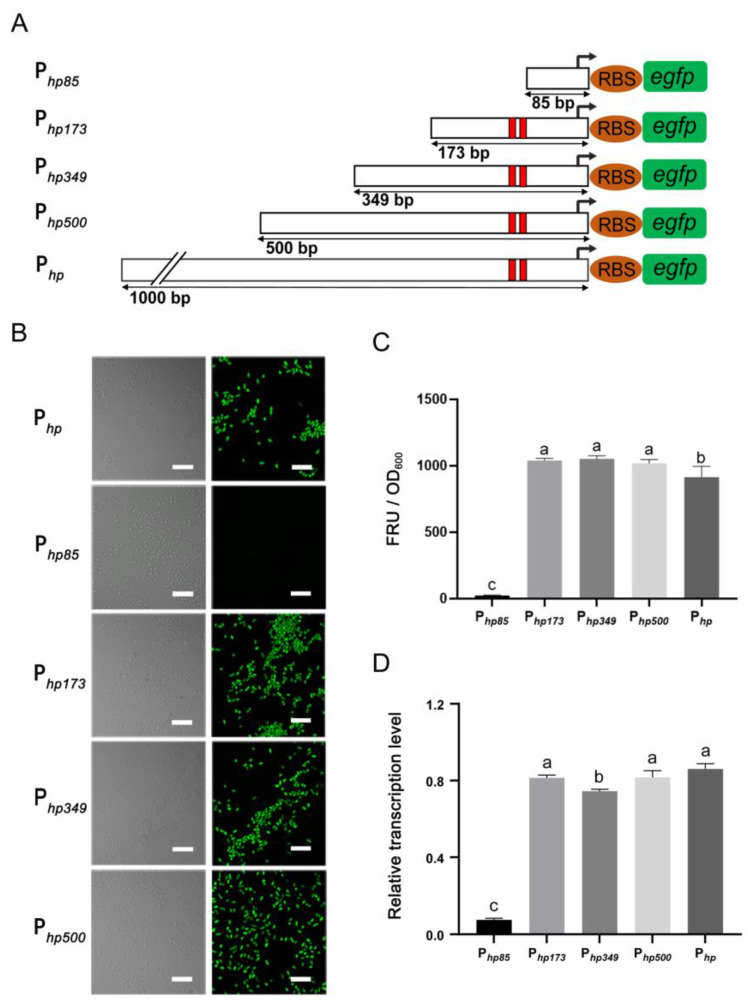
The determination of the minimum promoter. (**A**) Shortened diagram of the P1 promoter sequence. In the figure, 1000, 500, 349, 173 and 85 bp represent the upstream DNA lengths of the −35 region, corresponding to P*_hp_*, P*_hp500_*, P*_hp349_*, P*_hp173_* and P*_hp85_*, respectively. The red colors indicate −10 region and −35 region. (**B**) The fluorescence intensity of different shortened promoters was detected via confocal microscopy. (**C**) The fluorescence intensity was detected via confocal microscopy. (**D**) The transcriptional activity of each shortened promoter was detected via RT-qPCR. *recA* is the internal reference gene. The data represent the mean ± standard deviation of three replicates. Duncan multiple comparative analysis was used for statistical analysis. Different letters indicate significant differences. All images were obtained under the same detection conditions. Scale bar = 5 μm.

The promoter sequence length of 1000 bp may be too long to be universally applicable for prokaryotic gene expression. Based on the results of the core regions according to two prediction websites, which including the −35 region (Sextama frame), the −10 region (Pribnow frame) and the spacer region [32,33], the P*_hp_* promoter was shortened, as shown in Figure 2A. Four 5′-shortened promoters of P*_hp_* (P*_hp500_*, P*_hp349_*, P*_hp173_* and P*_hp85_*) were constructed, and their activities in JP2-270 were compared via fluorescence measurement and RT-qPCR after 24 h culture (Figure 2). Among them, P*_hp__173_* promoter was the shortest promoter with the core regions, whereas P*_hp__85_* promoter did not contain these elements. The results indicated that the activities of the shortened promoters of P*_hp500_*, P*_hp349_* and P*_hp173_* were not significantly different from those of the full-length P1 promoter. However, the activity of the P*_hp85_* promoter decreased dramatically compared to the P1 promoter, with only about 1% activity (Figure 2B–D). These data suggest that the 173 bp sequence upstream of the promoter P*_hp_* is critical for its activity. Thus, the minimal length of P*_hp_* can be reduced to 173 bp.

### 3.4. Detection of PRN in JP2-270

**Figure 3:**
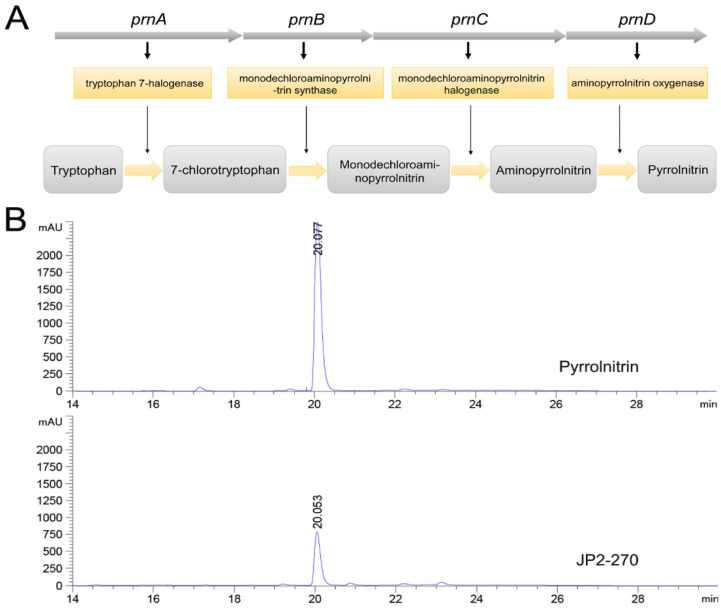
Fermentation crude extract analysis of JP2-270. (**A**) Biosynthetic steps for the synthesis of pyrrolnitrin. In the first step, *prnA* encodes tryptophan halogenase and catalyzes tryptophan chlorination to form 7-chlorotryptophan (7-CLT); in the second step, *prnB* encodes enzymes that catalyze the complex rearrangement and decarboxylation of 7-CLT from indole to phenylpyrrole to form monodechloroaminopyrrolnitrin (MDA); in the third step, *prnC* encodes an MDA halogenase that catalyzes the second chlorination of pyrrole ring position 3 to form amino-PRN; and in the final step, *prnD* encodes an enzyme that oxidizes the amino group of amino-PRN to a nitro group, forming PRN. (**B**) HPLC analysis of the JP2-270 fermentation crude extract and PRN standard. The peaks corresponding to 20.078 min and 20.053 min were pyrrolnitrin.

The biosynthetic pathway of PRN was previously reported (Figure 3A) [34]. Whole genome sequence analysis showed that the synthetic gene cluster of PRN was present in JP2-270, suggesting that JP2-270 has the potential to generate PRN. The PRN gene cluster in the JP2-270 genome contains four biosynthetic genes, namely, *prnA* (DM992_38835), *prnB* (DM992_38830), *prnC* (DM992_38825) and *prnD* (DM992_38820).

PRN has been proven to exhibit good antifungal activity on a variety of pathogenic fungi [8,12]. Therefore, JP2-270 was fermented to verify the presence of PRN in the antibacterial active substances produced by it, and the fermentation products were extracted. HPLC analysis showed that the fermentation crude extract of JP2-270 had a peak consistent with the retention time of the PRN standard (Figure 3B), indicating that JP2-270 can produce PRN.

### 3.5. Application of P_hp173_ to Improve Production of PRN

**Figure 4:**
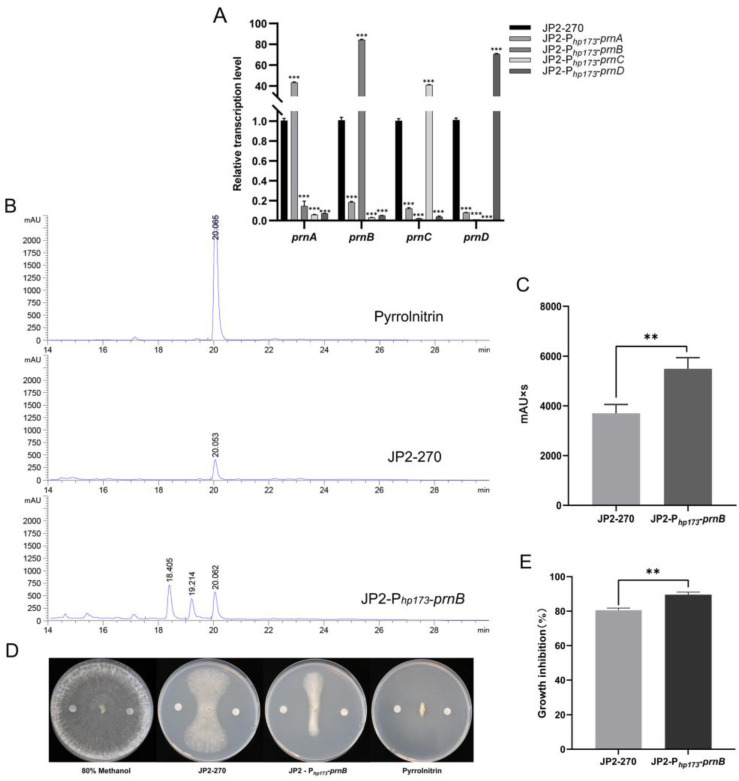
An analysis of the overexpression of PRN genes controlled by the strong promoter P*_hp173_* in *Burkholderia* sp. JP2-270. (**A**) The transcription levels of *prnA*, *prnB*, *prnC* and *prnD* under the control of the P*_hp173_* promoter in JP2-270 were detected via RT-qPCR. *recA* is the internal reference gene. The data represent the mean ± standard deviation of three replicates. Statistical analyses were performed and marked (***, *p* < 0.001). (**B**) HPLC analysis of fermentation crude extracts from *Burkholderia* sp. JP2-P*_hp173_*-*prnB* and JP2-270. The peaks corresponding to 20.067 min, 20.053 min and 20.062 min were PRN. (**C**) Comparison of peak area of PRN in *Burkholderia* sp. JP2-P*_hp173_*-*prnB* and JP2-270. The data represent three repeated mean values ± standard deviation. The *t-*test was used for statistical analysis and significance was marked (**, *p* < 0.01). (**D**) The inhibitory activity of fermentation crude extracts from *Burkholderia* sp. JP2-P*_hp173_*-*prnB* and JP2-270. The negative control was 80% methanol, and the PRN standard served as the positive control. (**E**) The histogram of growth inhibition of *Burkholderia* sp. JP2-P*_hp173_*-*prnB* and JP2-270. The data represent three repeated mean values ± standard deviation. The *t-*test was used for statistical analysis and significance was marked (**, *p* < 0.01).

In order to detect the activation effect of the screened endogenous strong promoter P*_hp173_ *on endogenous gene expression, P*_hp173_* was used as a promoter to construct four overexpression vectors of PRN synthetic genes. The transcriptional levels of these four genes were detected via RT-qPCR, and the results showed that their transcriptional levels were significantly improved by 40–80 times (Figure 4A). This suggests that the endogenous promoter P*_hp173_* can efficiently drive the expression of endogenous genes in JP2-270.

To investigate the effect of overexpressing PRN synthesis genes under the control of the endogenous strong promoter P*_hp173_* on the accumulation of JP2-270 metabolites, the fermentation crude extracts of four overexpressed recombinant strains were analyzed via HPLC. No increase in PRN production was detected in the three overexpressing recombinant strains of *prnA*, *prnC* and *prnD*, but the yield of PRN was 1.46 times that of the wild type in the *prnB*-overexpressing strain, according to the calculation of the peak area in HPLC analysis (Figure 4B,C). Notably, the crude extraction of JP2-270 inhibited the growth of *R. solani* GD118 by 80.37%, and the crude extraction of *prnB*-overexpressing strain inhibited the growth of *R. solani* GD118 by 89.24%. These results indicate that P*_hp173_* may promote efficient gene expression and increase the production of PRN in JP2-270, which had strong inhibitory activity on *R. solani* GD118.

## 4. Discussion

In recent years, *Burkholderia* has attracted increasing attention because of its outstanding potential in biological control. More than 20 closely related species of *Burkholderia* genus are divided into the *Burkholderia cepacia* complex (Bcc) [35]. Bcc often colonize the rhizosphere of some plant species [36]. Some of them have shown an effective ability to inhibit many plant diseases and promote plant growth. The *Burkholderia nodosa* strain isolated from the soil in Kalimantan, Indonesia, has been shown to exhibit significant inhibition against bacterial wilt and blight [37]. The endophytic bacteria *Burkholderia ambifaria* isolated from wheat plants have antagonistic activity against *Rhizoctonia cerealis* and can be used as a new, effective biological control agent for wheat sharp eyespot disease [38]. *Burkholderia* sp. JP2-270, isolated from rice rhizosphere soil, has demonstrated good inhibition of rice sheath blight [39].

RNA-Seq is a common method used in promoter engineering [40]. In this study, of the fifty candidate promoters we screened, only nine were able to generate a significant fluorescence signal under the microscope (Figure 1A). The order of transcription levels of the nine promoters ranked by RT-qPCR does not correspond to that ranked via the TPM values in RNA-seq, as reported in other studies [41]. This may be because the transcription levels of endogenous promoters can be influenced by other neighboring genes and regulatory sequences in the genome. In addition, certain promoters with higher transcriptional levels showed weaker fluorescence, such as P9 and P37, which is consistent with the findings reported in other studies, possibly due to poor compatibility with RBS sequences added downstream (Figure 1) [42].

The promoter sequence of prokaryotes is generally short, and its typical conserved regions include the transcription initiation site (TSS), the −35 region, the −10 region and the spacer region [31,32]. The 500 bp promoter Prsp_7571 was shortened according to the distant regions of −10 and −35 from far to near, resulting in a 167 bp promoter shortened with transcriptional activity 3–4 times higher than that of the control promoter *tac* [43]. In our study, the core components and positions of the −10 and −35 regions of the P*_hp_* promoter were determined, and a shorter length (173 bp) of the P*_hp_* promoter was experimentally determined. At the same time, we also tested a shortened promoter with a length of 85 bp without core components, and the activity was reduced by more than 90% compared to the original length P*_hp_* promoter (Figure 2).

PRN was first discovered in 1964, and its biosynthetic pathway was subsequently illustrated [34]. In this study, we focused on genes that can lead to the accumulation of the product. The screened endogenous strong promoter was used to overexpress key genes in biosynthesis to increase the yield of PRN in JP2-270. When the four PRN genes in JP2-270 were individually overexpressed, the transcription level of each gene was increased by 40–80 times (Figure 4A). However, an increase in the yield of PRN was detected only in *Burkholderia* sp. JP2-P*_hp173_*-*prnB* (Figure 4B), which suggests that in JP2-270, only the overexpressed *prnB* contributes to the accumulation of PRN.

HPLC analysis of *Burkholderia* sp. JP2-P*_hp173_*-*prnB* showed that the overexpression of *prnB* also led to the appearance of two other peaks, which were absent in wild-type JP2-270 (Figure 4B). Because the enzyme encoded by the *prnB* gene can catalyze the complex rearrangement and decarboxylation of pyrrole rings, the *prnB* gene product can either convert 7-CLT into MDA in the presence of 7-CLT, or directly use tryptophan as a substrate to generate aminophenylpyrrole [44]. Thus, the overexpression of *prnB* may lead to the accumulation of MDA and APP. Further analysis of the two peaks in Figure 4B is important to improve our understanding of the PRN biosynthetic pathway.

## 5. Conclusions

In summary, an endogenous strong promoter, P*_hp_*, was screened from the JP2-270 genome via RNA-Seq analysis, combined with fluorescence detection and RT-qPCR identification. The promoter P*_hp_* was shortened at different lengths to produce a shorter endogenous strong promoter, P*_hp173_*, containing the active region. P*_hp173_* was further used to overexpress four endogenous PRN biosynthetic genes, namely, *prnA*, *prnB*, *prnC* and *prnD,* in JP2-270. The results demonstrate that the promoter can improve the transcription levels of endogenous genes. At the same time, the overexpression of the *prnB* gene also increased the production of the JP2-270 metabolite PRN. Our results show that the promoter screened in this study, P*_hp__173_*, can improve the production of secondary metabolites in JP2-270.

## Figures and Tables

**Table 1:** Plasmids and strains used in this study.

Plasmids or Strains	Characteristics	Sources
Plasmids		
pBBR1MCS-2	Broad-host cloning vector, Km^R^	Kovach et al., 1995 [22]
pBBR1MCS-Cm	Cm^R^, pBBR1MCS-2 derived, shuttle vector of JP2-270, used for expressing of genes	This study
pBBR1MCS-Cm-P1-*eGFP*	Cm^R^, pBBR1MCS-Cm derivative containing P1 promoter and *eGFP* gene, shuttle vector of JP2-270, used for expressing of *eGFP*	This study
pBBR1MCS-Cm-P*_hp85_*-*eGFP*	pBBR1MCS-Cm-P1-*eGFP* derived, P1 promoter replaced by the P*_hp85_* promoter	This study
pBBR1MCS-Cm-P*_hp173_*-*eGFP*	pBBR1MCS-Cm-P1-*eGFP* derived, P1 promoter replaced by the P*_hp173_* promoter	This study
pBBR1MCS-Cm-P*_hp349_*-*eGFP*	pBBR1MCS-Cm-P1-*eGFP* derived, P1 promoter replaced by the P*_hp349_* promoter	This study
pBBR1MCS-Cm-P*_hp500_*-*eGFP*	pBBR1MCS-Cm-P1-*eGFP* derived, P1 promoter replaced by the P*_hp500_* promoter	This study
pBBR1MCS-Cm-P*_hp173_*-*prnB*	pBBR1MCS-Cm-P*_hp173_*-*eGFP* derived, *eGFP* replaced by *prnB*, used for overexpressing of *prnB*	This study
Strains		
*E. coli* TOP10	Used for plasmid construction	Purchased from EASY-DO
*Burkholderia* sp. JP2-270	Wild type	Stored in lab
*Burkholderia* sp. JP2-P*_hp173_*-*prnB*	JP2-270 derivative containing pBBR1MCS-Cm-P*_hp173_*-*prnB*; Cm^R^	This study
*Rhizoctonia solani* GD118	Type strain	Donated by Shiwen Huang’s lab from CNRRI

## Data Availability

The original contributions presented in the study are included in the article/Supplementary Material, further inquiries can be directed to the corresponding author.

## Supplementary Materials

The following supporting information can be downloaded at: https://www.mdpi.com/article/10.3390/microorganisms12091818/s1, Figure S1: Growth curve of *Burkholderia* sp. JP2-270; Table S1: Primer sequences used in this study; Figure S2: TPM distribution of transcripts of all genes in sequencing samples; Table S2: The top 50 genes ranked by transcriptional level based on their TPM values in RNA-seq analysis (16 h); Table S3: The top 50 genes ranked by transcriptional level based on their TPM values in RNA-seq analysis (24 h); Table S4: The top 50 genes ranked by transcriptional level based on their TPM values in RNA-seq analysis (48 h); Table S5: 50 highly expressed genes for promoter cloning; Table S6: Promoters sequences identified in this study.
