# Supplementary material for: Identification of an Endogenous Strong Promoter in Burkholderia sp. JP2-270
Source: Microorganisms. 2024 Sep 2;12(9):1818. doi: 10.3390/microorganisms12091818 (PMC11434214; doi:10.3390/microorganisms12091818)
Supplement: Supplementary file 1 [file microorganisms-12-01818-s001.zip › Supplementary_Figure.pdf]

Supplementary figure

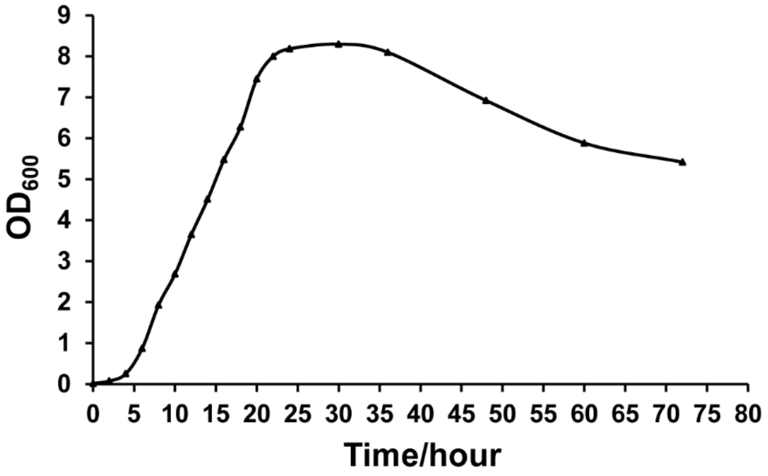

Figure S1. Growth curve of *Burkholderia* sp. JP2-270.

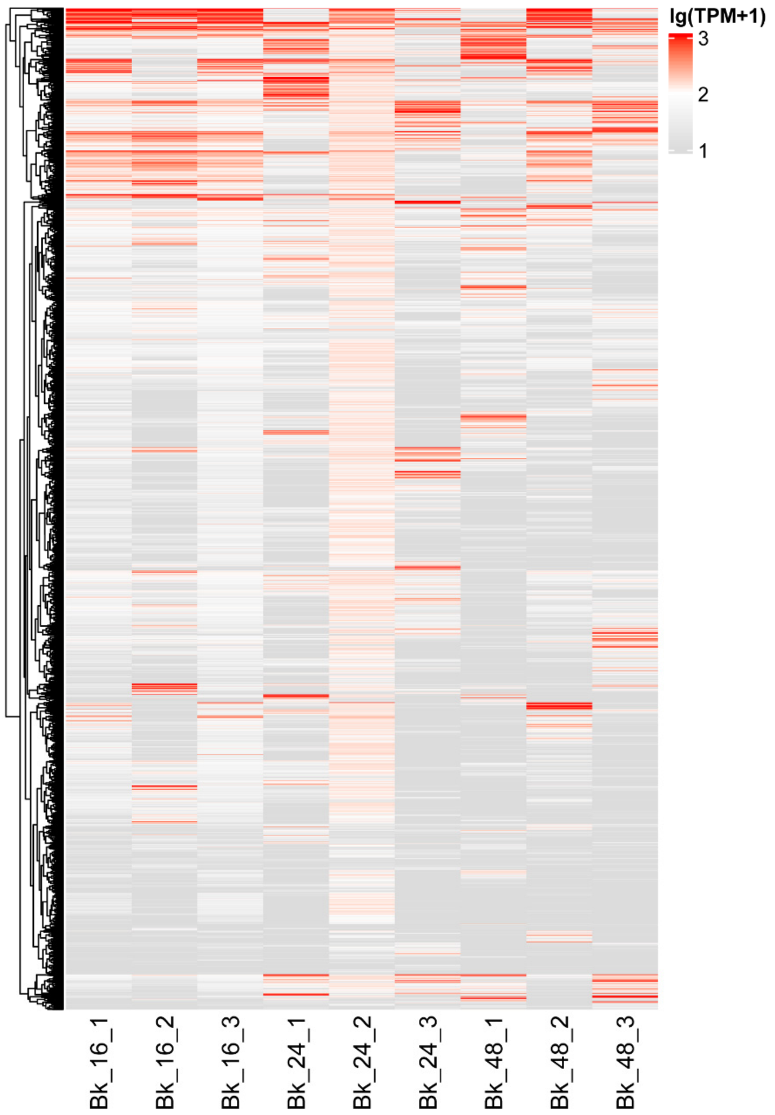

Figure S2. TPM distribution of transcripts of all genes in sequencing samples.
